# Degree of mosaicism in trophectoderm does not predict pregnancy potential: a corrected analysis of pregnancy outcomes following transfer of mosaic embryos

**DOI:** 10.1186/s12958-018-0322-5

**Published:** 2018-01-26

**Authors:** Vitaly A. Kushnir, Sarah K. Darmon, David H. Barad, Norbert Gleicher

**Affiliations:** 1 Center for Human Reproduction, 21 East 69th Street, New York, NY 10021 USA; 2 Department of Obstetrics and Gynecology, Wake Forest School of Medicine, Winston-Salem, NC USA; 3 Foundation for Reproductive Medicine, New York, NY USA; 4 Stem Cell Biology and Molecular Embryology Laboratory, The Rockefeller University, New York, NY USA; 5 Department of Obstetrics and Gynecology, University of Vienna School of Medicine, Vienna, Austria

**Keywords:** In vitro fertilization, Preimplantation genetic diagnosis, Preimplantation genetic screening, Aneuploidy, Embryo selection, Next-generation sequencing

## Abstract

**Background:**

Preimplantation genetic screening (PGS) is increasingly utilized as an adjunct procedure to IVF. Recently healthy euploid live birth were reported following transfer of mosaic embryos. Several recent publications have surmised that the degree of trophectoderm (TE) mosaicism in transferred embryos is predictive of ongoing pregnancy and miscarriage rates.

**Methods:**

This is a corrected analysis of previously published retrospective data on vitro fertilization (IVF) cycle outcomes involving replacement of 143 mosaic and 1045 euploid embryos tested by PGS, utilizing high-resolution next-generation sequencing (NGS) of TE and determination of percentages of mosaicism. Receiver operating curves (ROCs) and measurement of area under the curve (AUC) were used to evaluated the accuracy of the predictor variable, proportion of aneuploid cells in a TE biopsy specimen, with IVF outcomes, ongoing pregnancy and miscarriage rates.

**Results:**

Confirming findings of the previously published report we also found higher ongoing pregnancy rates (63.3% vs. 39.2%) and lower miscarriage rates (10.2% vs. 24.3%) with euploid embryo transfers than with mosaic embryo transfer. There, however, were no significant differences in ongoing pregnancy or miscarriage rates among mosaic embryo transfers at any threshold of aneuploidy. Based on AUC, TE biopsies predicted ongoing pregnancy for euploid, as well as mosaic embryos, in a range of 0.50 to 0.59 and miscarriage in a range from 0.50 to 0.66

**Conclusions:**

Degree of TE mosaicism was a poor predictor of ongoing pregnancy and miscarriage.

**Electronic supplementary material:**

The online version of this article (10.1186/s12958-018-0322-5) contains supplementary material, which is available to authorized users.

## Background

Preimplantation genetic screening (PGS), now renamed by some as preimplantation genetic testing for aneuploidy (PGT-A), is increasingly utilized as ad-on to vitro fertilization (IVF). The original hypothesis behind PGS utilization was “*aneuploidy screening as a means to increase pregnancy rates, decrease loss rates, and establish transfer order*” [1]. Because of growing recognition that trophectoderm (TE) mosaicism is a common finding [2], the Preimplantation Genetic Diagnosis International Society (PGDIS) recently recommended a radical overhaul of testing methodologies and reporting of test results [3].

Since diagnostic platforms, like array comparative genome hybridization (aCGH), single-nucleotide polymorphism array, and quantitative polymerase chain reaction (qPCR) lack capacity to detect mosaicism in a single TE biopsy, next-generation sequencing (NGS), which currently detects mosaicism in excess of 20%, is the only technique recommended by PGDIS [3]. Utilizing NGS, embryos with less than 20% aneuploidy in the TE sample are, therefore, considered euploid; while those between 20 and 80% are reported as mosaic, and those over 80% as truly aneuploid. Moreover, PGDIS guidelines suggest that embryos designated as euploid can be freely transferred, while embryos designated as aneuploid should not be transferred and, therefore, discarded [3].

Finally, mosaic embryos, at 20–80% range aneuploidy in the TE sample, may be potentially transferred, though the PGDIS notes that such transfers be performed with caution and only in absence of euploid embryos. Moreover, the society suggested an empirical hierarchy for such transfers, based on the specific aneuploidies reported in embryos [3].

A group of investigators at the 2016 World Congress on Controversies in Preconception, Preimplantation, Prenatal Genetic Diagnosis Meeting in Barcelona reached similar conclusions, advising to prioritize transfer of mosaic embryos with lower levels (20–40%) of aneuploidy in the TE sample over those with higher levels (40–70%), and defining any embryo biopsy with more than 70% aneuploidy in its TE as aneuploid and, therefore, as not transferrable [4].

Both sets of guidelines, however, were lacking robust published clinical data in support, as are usually required for clinical diagnostic testing [5, 6]. This why a recent publication by a multi-center conglomerate of PGS reference laboratories and referring IVF centers, offering a first large data sets of clinical outcomes following transfer of mosaic blastocyst in accordance with PGDIS criteria and utilizing NGS [7], has to be viewed as a defining moment. Until these recent publications, only three groups have reported IVF cycle outcomes after transfers of mosaic embryos in a small number of cases [8–10].

Since the manuscripts by (Munné et al. 2017; Fragouli et al. 2017) did not use standard statistical methods to assess predictive values for different degrees of mosaicism detected in TE biopsies, we reanalyzed the raw data reported by the authors. As this study will demonstrate, our analysis contradicts the conclusions of Munné et al. that 40% mosaicism represents a significant differentiation point. Indeed, our analysis did not find significant predictability at any level of mosaicism between 20 and 80%.

## Methods

A detailed description of patient factors and molecular methods is available in the original publications, which served as data sources for our study [7, 11]. The data for 143 NGS-tested mosaic embryos that were transferred, were extracted from Additional file 1: Table S3 in the publication by Munné et al. [7]. To assess whether the degree of mosaicism at different ages affected ongoing pregnancy rates, these data were stratified for female ages < 38 and ≥38 years.

Data for the comparison group came from for female age well matched controls from the same publication, who had undergone transfers of 1045 euploid embryos, as determined by NGS with < 20% aneuploid cells in the TE sample [7]. This control group could not be age-stratified since only aggregate data (rather than embryo level data) were provided in the source publication.

Receiver operating characteristic curves (ROCs) and measurements of area under the curve (AUC) were used to evaluated accuracy of the predictor variable, proportion of abnormal cells in a TE biopsy specimen (i.e., percentage mosaicism) with IVF outcomes, including ongoing pregnancy and miscarriage rates.

**Fig. 1:**
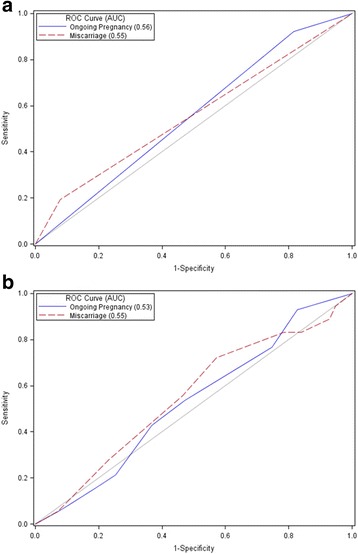
Receiver operating characteristic (ROC) curves for ongoing pregnancy and miscarriage. **a** Analysis for binary classification euploid (< 20% aneuploid cells) vs. mosaic embryos (20% to 80% aneuploid cells) based on trophectoderm sample. **b** Various thresholds of aneuploidy within the cohort of mosaic embryos ranging from 20% to 80% in 10% increments. Predictor variable: proportion of abnormal cells in a trophectoderm biopsy specimen. Outcomes: ongoing pregnancy and miscarriage. Area under the curve (AUC) of 0.50 denotes the screening test as having no predictive ability while an AUC of 1.0 denotes an ideal screening test

The analysis was then performed for binary variables euploid (< 20% aneuploid cells) vs. mosaic embryos, and for various thresholds of aneuploidy, ranging from 20% to 80% in 10% increments, as detailed in Table 1 and Fig. 1.

**Table 1:** Predictive ability of NGS trophectoderm biopsy for ongoing pregnancy based on proportion of abnormal cells in the specimen

	Euploid < 20% Abnormal		Mosaic ≥20% to ≤80% Abnormal		*p*-value	AUC
Ongoing Pregnancy	661/1045	63.3%	56/143	39.2%	< 0.0001	0.55
	Mosaic ≥20% to < 30% Abnormal		Mosaic ≥30% to ≤80% Abnormal		*p*-value	AUC
Age	Ongoing Pregnancy					
< 38	12/27	44.4%	22/61	36.1%	0.46	0.54
≥ 38	1/8	12.5%	21/46	45.7%	0.08	0.59
All Ages	13/35	37.1%	43/108	39.9%	0.78	0.51
	Mosaic ≥20% to < 40% Abnormal		Mosaic ≥40% to ≤80% Abnormal		*p*-value	AUC
Age	Ongoing Pregnancy					
< 38	22/61	36.1%	12/27	44.4%	0.46	0.54
≥ 38	10/25	40.0%	12/29	41.4%	0.92	0.51
All Ages	32/87	36.8%	24/56	42.9%	0.47	0.53
	Mosaic ≥20% to < 50% Mosaic		Mosaic ≥50% to ≤80% Abnormal		*p*-value	AUC
Age	Ongoing Pregnancy					
< 38	30/78	38.5%	4/10	40.0%	0.93	0.50
≥ 38	17/38	44.7%	5/16	31.3%	0.36	0.56
All Ages	47/117	40.2%	9/26	34.6%	0.60	0.52
	Mosaic ≥20% to < 60% Abnormal		Mosaic ≥60% to ≤80% Abnormal		*p*-value	AUC
Age	Ongoing Pregnancy					
< 38	34/86	39.5%	0/2	0.0%	0.26	0.52
≥ 38	18/44	40.9%	4/10	40.0%	0.96	0.50
All Ages	52/131	39.7%	4/12	33.3%	0.67	0.51
	Mosaic ≥20% to < 70% Abnormal		Mosaic ≥70% to ≤80% Abnormal		*p*-value	AUC
Age	Ongoing Pregnancy					
< 38	34/87	39.1%	0/1	0.0%	0.42	0.51
≥ 38	19/46	41.3%	3/8	37.5%	0.84	0.51
All Ages	53/134	39.6%	3/9	33.3%	0.71	0.51

AUC: area under the curve

Comparisons between groups were made using chi-square tests. Statistical analyses were preformed using SAS version 9.4. A *p*-value < 0.05 was considered statistically significant.

Since all here addressed data were already published, publicly available, and cannot be utilized to identify individual patients, this study qualified for exemption from IRB approval.

## Results

Table 1 shows the ability of TE biopsies, utilizing NGS, to predict ongoing pregnancy based on proportion of abnormal cells in each biopsy specimen. Here we confirm the findings of Munné et al. [7] that ongoing pregnancy rates were, seemingly, significantly higher following euploid embryo transfer (i.e., biopsies with < 20% aneuploidy) than mosaic embryo transfer (biopsies with 20–80% aneuploidy). Our calculation yielded a slightly lower ongoing pregnancy rate in the mosaic group than Munné et al., who reported 57 ongoing and delivered pregnancies but presented embryo level data for only 56.

Table 1 demonstrates no significant differences in ongoing pregnancy rates among mosaic transfers at any threshold of aneuploidy. Moreover, the table demonstrates the AUC for all comparisons ranges from 0.50 to 0.59, indicating poor predictive ability of TE biopsies for ongoing pregnancies.

Table 2 shows the predictive ability of TE biopsies, utilizing NGS, to predict miscarriages based on proportion of abnormal cells in a single biopsy sample. Once again we confirm the findings of Munné et al. [7] that miscarriage rates were significantly lower following euploid embryo transfer than with mosaic embryo transfer. However, the table also demonstrates that the AUC is only 0.56, again indicting very poor predictive validity of a TE biopsy for miscarriages. Moreover, analysis of miscarriage risk among mosaic embryo transfers, did not find increased risk based on any threshold of aneuploidy.

**Table 2:** Predictive ability of NGS trophectoderm biopsy for miscarriage based on proportion of abnormal cells in the specimen

	Euploid < 20% Abnormal		Mosaic ≥20% to ≤80% Abnormal		*p*-value	AUC
Miscarriage	75/736	10.2%	18/74	24.3%	0.0003	0.56
	Mosaic ≥20% to < 30% Abnormal		Mosaic ≥30% to ≤80% Abnormal		*p*-value	AUC
Age	Miscarriage					
< 38	5/17	29.4%	9/31	29.0%	0.69	0.50
≥ 38	0/1	0.0%	4/25	16.0%	0.66	0.52
All Ages	5/18	27.8%	13/56	23.2%	0.69	0.52
	Mosaic ≥20% to < 40% Abnormal		Mosaic ≥40% to ≤80% Abnormal		*p*-value	AUC
	Miscarriage					
< 38	11/33	33.3%	3/15	20.0%	0.35	0.57
≥ 38	2/12	16.7%	2/14	14.3%	0.87	0.52
All Ages	13/45	28.9%	5/29	17.2%	0.25	0.58
	Mosaic ≥20% to < 50% Mosaic		Mosaic ≥50% to ≤80% Abnormal		*p*-value	AUC
Age	Miscarriage					
< 38	13/43	30.2%	1/5	20.0%	0.63	0.52
≥ 38	2/19	10.5%	2/7	28.6%	0.26	0.64
All Ages	15/62	24.2%	3/12	25.0%	0.95	0.50
	Mosaic ≥20% to < 60% Abnormal		Mosaic ≥60% to ≤80% Abnormal		*p*-value	AUC
Age	Miscarriage					
< 38	14/48	29.2%	0/0	0.0%	–	–
≥ 38	2/20	10.0%	2/6	33.3%	0.16	0.66
All Ages	16/68	23.5%	2/6	33.3%	0.59	0.59
	Mosaic ≥20% to < 70% Abnormal		Mosaic ≥70% to ≤80% Abnormal		*p*-value	AUC
Age	Miscarriage					
< 38	14/48	29.2%	0/0	0.0%	–	–
≥ 38	3/22	13.6%	1/4	25.0%	0.56	0.56
All Ages	17/70	24.3%	1/4	25.0%	0.97	0.50

AUC: area under the curve

Figure 1a demonstrates ROC curves for ongoing pregnancies and miscarriages based on binary classification of embryos as either euploid or mosaic. Figure 1b shows ROC curves for ongoing pregnancies and miscarriages based on various thresholds of aneuploidy (in 10% increments, ranging from 20% to 80%) within the group of mosaic embryos. Both figures show a null line for reference with an AUC of 0.50 which denotes when a screening test has no predictive ability.

## Discussion

Our analysis confirms the previously published report which found statistically higher ongoing pregnancy rates (63.3% vs. 39.2%) and lower miscarriage rates (10.2% vs. 24.3%) with euploid embryo transfers than with mosaic embryo transfer. These findings are also consistent with a recent report by Spinella et al. [12]. However, our analysis demonstrates with ROC curves that PGS, at any threshold level of mosaicism, up to 80% aneuploidy in a single TE biopsy as determined by NGS, has poor predictive value as a screening test for an individual embryo’s ability to establish ongoing pregnancy or lead to miscarriage. Moreover, given the observed ongoing pregnancy and miscarriage rates it appears likely that in many cases aneuploidy within the TE of embryos represents a physiologic variant rather than a pathological condition. This conclusion is supported by a recent in depth literature review: “*Mosaicism in Preimplantation Human Embryos: When Chromosomal Abnormalities Are the Norm*” [13].

Commonly utilized screening tests in reproductive medicine, such as mammograms and PAP smears for cancer screening [14], while themselves not ideal screening tests, still demonstrate far more robust clinical performance than here presented PGS data. This study reinforces previously raised concerns about the biological premise and clinical effectiveness of PGS [15–18]. It is our view that to ensure optimal outcomes for patients the reproductive medicine community should hold commercial PGS tests to the same standards as other screening tests commonly utilized in the field. Therefore, in our opinion commercial laboratories which market PGS tests should seek regulatory approval for these tests [19].

Based on the original data set published by Munné et al. [7], our analysis may, indeed, actually still overestimate the benefits of PGS in this patient population. In the original data set, the authors indicated that 133 out of 143 mosaic embryo transfers were single embryo transfers, while 10 were double embryo transfers (such data were not provided for the control group of patients undergoing euploid embryo transfer). A higher proportion of double embryo transfers in the euploid embryo group, could, therefore, hypothetically explain the higher ongoing pregnancy rate and lower miscarriage rates observed in this group.

It is also likely that patients who produce euploid embryos have better prognoses and/or less severe underlying infertility than those who only produce mosaic embryos. This point is consistent with several recent studies which have found a correlation between proportion of aneuploid blastocysts and diminished ovarian reserve while controlling for patient age [20–22]. Finally, the original dataset does not specify the number of treated patients nor the number of tested embryos per patient in either group. It reflects only patients who reached embryo transfer, thereby excluding poorer prognosis patients who started IVF with intention of PGS but did not have any transferrable embryos following extended embryo culture, TE biopsy, cryopreservation, and thawing. The patient selection is, therefore, biased toward better prognosis patients, as previously documented in national registry data in patients undergoing IVF with PGS [23]. Additional limitations include reporting of ongoing pregnancy rates rather than live birth rates and lack of data on the health of infants conceived from mosaic embryos.

We conclude that the degree of TE mosaicism is a poor predictor of ongoing pregnancy and miscarriage. Moreover, in our ROC analysis PGS demonstrates poor clinical effectiveness for a routine screening test and, therefore, should not be routinely offered as an ad-on to IVF. Until efficacy, safety, and cost effectiveness of PGS are established it, therefore, should only be offered as an experimental test under study conditions and with appropriate informed consent.

## Additional file

Additional file 1: Data for 143 NGS-tested mosaic embryos that were transferred; extracted from Table S3 in the publication by Munné et al. [7]. (XLSX 17 kb)

## Abbreviations

aCGH: Array comparative genome hybridization
AUC: Area under the curve
CLIA: Clinical Laboratory Improvement Amendments
FDA: Food and Drug Administration
IVF: In vitro fertilization
LDTs: Laboratory-developed tests
NGS: Next-generation sequencing
PGDIS: Preimplantation Genetic Diagnosis International Society
PGS: Preimplantation genetic screening
qPCR: Quantitative polymerase chain reaction
ROCs: Receiver operating curves
TE: Trophectoderm

## References

[1] Franasiak JM, Forman EJ, Hong KH, Werner MD, Upham KM, Treff NR, Scott RT (2014). The nature of aneuploidy with increasing age of the female partner: a review of 15,169 consecutive trophectoderm biopsies evaluated with comprehensive chromosomal screening. Fertil Steril.

[2] Sachdev NM, Maxwell SM, Besser AG, Grifo JA (2017). Diagnosis and clinical management of embryonic mosaicism. Fertil Steril.

[3] PGDIS position statement on chromosome mosaicism and preimplantation aneuploidy testing at the blastocyst stage. [http://www.pgdis.org/docs/newsletter_071816.html].

[4] Controversies in Preconception, Preimplantation, and Prenatal Genetic Diagnosis. CoGen position statement on chromosomal mosaicism detected inpreimplantation blastocyst biopsies. [https://www.ivf-worldwide.com/index.php?option=com_content&view=article&id=733&Itemid=464].

[5] Standards for Guideline Development - US Preventive Services Task Force [https://www.uspreventiveservicestaskforce.org/Page/Name/standards-for-guideline-development].

[6] Standards for Developing Trustworthy Clinical Practice Guidelines : Health and Medicine Division. [http://www.nationalacademies.org/hmd/Reports/2011/Clinical-Practice-Guidelines-We-Can-Trust/Standards.aspx].

[7] Munné S, Blazek J, Large M, Martinez-Ortiz PA, Nisson H, Liu E, Tarozzi N, Borini A, Becker A, Zhang J, Maxwell S, Grifo J, Babariya D, Wells D, Fragouli E. Detailed investigation into the cytogenetic constitution and pregnancy outcome of replacing mosaic blastocysts detected with the use of high-resolution next-generation sequencing. Fertil Steril. 2017;108(1):62-71.e8. 10.1016/j.fertnstert.2017.05.002 28579407

[8] Greco E, Minasi MG, Fiorentino F (2015). Healthy babies after intrauterine transfer of mosaic Aneuploid blastocysts. N Engl J Med.

[9] Gleicher N, Vidali A, Braverman J, Kushnir VA, Barad DH, Hudson C, Wu YG, Wang Q, Zhang L, Albertini DF (2016). International PGS consortium study group: accuracy of preimplantation genetic screening (PGS) is compromised by degree of mosaicism of human embryos. Reprod Biol Endocrinol.

[10] Lledó B, Morales R, Ortiz JA, Blanca H, Ten J, Llácer J, Bernabeu R (2017). Implantation potential of mosaic embryos. Syst Biol Reprod Med.

[11] Fragouli E, Alfarawati S, Spath K, Babariya D, Tarozzi N, Borini A, Wells D (2017). Analysis of implantation and ongoing pregnancy rates following the transfer of mosaic diploid-aneuploid blastocysts. Hum Genet.

[12] Spinella F, Fiorentino F, Biricik A, Bono S, Ruberti A, Cotroneo E, Baldi M, Cursio E, Minasi MG, Greco E. Extent of chromosomal mosaicism influences the clinical outcome of in vitro fertilization treatments. Fertil Steril. 2017; 10.1016/j.fertnstert.2017.09.025 29191449

[13] McCoy RC (2017). Mosaicism in preimplantation human embryos: when chromosomal abnormalities are the norm. Trends Genet.

[14] Maxim LD, Niebo R, Utell MJ (2014). Screening tests: a review with examples. Inhal Toxicol.

[15] Gleicher N, Kushnir VA, Barad DH (2014). Preimplantation genetic screening (PGS) still in search of a clinical application: a systematic review. Reprod Biol Endocrinol.

[16] Mastenbroek S, Repping S (2014). Preimplantation genetic screening: back to the future. Hum Reprod.

[17] Orvieto R. Preimplantation genetic screening- the required RCT that has not yet been carried out. Reprod Biol Endocrinol. 2016;14(35). 10.1186/s12958-016-0171-z PMC4921019 27342051

[18] Gleicher N, Metzger J, Croft G, Kushnir VA, Albertini DF, Barad DH. A single trophectoderm biopsy at blastocyst stage is mathematically unable to determine embryo ploidy accurately enough for clinical use. Reprod Biol Endocrinol. 2017;15. 10.1186/s12958-017-0251-8 PMC5408377 28449669

[19] The Public Health Evidence for FDA Oversight of Laboratory Developed Tests: 20 Case Studies. [https://www.fda.gov/MedicalDevices/ProductsandMedicalProcedures/InVitroDiagnostics/LaboratoryDevelopedTests/default.htm].

[20] Katz-Jaffe MG, Surrey ES, Minjarez DA, Gustofson RL, Stevens JM, Schoolcraft WB (2013). Association of abnormal ovarian reserve parameters with a higher incidence of aneuploid blastocysts. Obstet Gynecol.

[21] La Marca A, Minasi MG, Sighinolfi G, Greco P, Argento C, Grisendi V, Fiorentino F, Greco E (2017). Female age, serum antimüllerian hormone level, and number of oocytes affect the rate and number of euploid blastocysts in in vitro fertilization/intracytoplasmic sperm injection cycles. Fertil Steril.

[22] Shahine LK, Marshall L, Lamb JD, Hickok LR (2016). Higher rates of aneuploidy in blastocysts and higher risk of no embryo transfer in recurrent pregnancy loss patients with diminished ovarian reserve undergoing in vitro fertilization. Fertil Steril.

[23] Kushnir VA, Darmon SK, Albertini DF, Barad DH, Gleicher N (2016). Effectiveness of in vitro fertilization with preimplantation genetic screening: a reanalysis of United States assisted reproductive technology data 2011-2012. Fertil Steril.

